# Dynamic Responses in a Plant-Insect System to Fertilization by Cormorant Feces

**DOI:** 10.3390/insects6020419

**Published:** 2015-04-24

**Authors:** Gundula Kolb, Peter A. Hambäck

**Affiliations:** Department of Ecology, Environment and Plants Sciences, Stockholm University, Stockholm SE-106 91, Sweden; E-Mail: gundula.kolb@gmail.com

**Keywords:** fertilization, *Lythrum salicaria*, *Galerucella*, cormorants

## Abstract

Theoretical arguments suggest that increased plant productivity may not only increase consumer densities but also their fluctuations. While increased consumer densities are commonly observed in fertilization experiments, experiments are seldom performed at a spatial and temporal scale where effects on population fluctuations may be observed. In this study we used a natural gradient in soil fertility caused by cormorant nesting. Cormorants feed on fish but defecate on their nesting islands. On these islands we studied soil nutrient availability, plant nutrient content and the density of *Galerucella* beetles, main herbivores feeding on *Lythrum salicaria*. In a common garden experiment, we followed larval development on fertilized plants and estimated larval stoichiometry. Soil nutrient availability varied among islands, and several cormorant islands had very high N and P soil content. Plant nutrient content, however, did not vary among islands, and there was no correlation between soil and plant nutrient contents. Beetle densities increased with plant nutrient content in the field study. However, there was either no effect on temporal fluctuations in beetle density or that temporal fluctuations decreased (at high P). In the common garden experiment, we found limited responses in either larval survival or pupal weights to fertilization. A possible mechanism for the limited effect of fertilization on density fluctuations may be that the distribution of *L. salicaria* on nesting islands was restricted to sites with a lower N and P content, presumably because high N loads are toxic.

## 1. Introduction

An increased soil nutrient availability affects plant nutrient content, stoichiometry and biomass, and therefore indirectly the resource availability and quality to herbivorous insects [1]. Many studies also show that insect performance is increased on nutrient rich plants and that insect numbers are typically higher in areas with nutrient rich soils [2,3,4], even though this response varies between taxa in a community [5,6]. Beside the effects on herbivore numbers, theory suggests that a high nutrient availability should also destabilize population dynamics [7,8,9]. Population dynamics are assumedly destabilized because high resource availability may allow for very high population growth rates in years when other conditions are suitable. In predator-prey system, theory suggests that time lags in the predator numerical response would similarly cause prey populations to fluctuate more at high productivity. The probability of unstable dynamics may however decrease due to interference among consumers [10], or spatial heterogeneity [11].

Many empirical studies have examined the effect from varying resource availability on consumer number and dynamics. These studies, however, are mostly done at a spatial and temporal scale where observations of population fluctuations are not relevant measures of population dynamics. There are a few exceptions involving model communities of limnic systems, and these studies provide little support to the destabilizing effect of nutrient enrichment. For instance, McCauley *et al*. [7] found that the presence of inedible prey reduced the destabilizing effect of nutrient enrichment on *Daphnia* dynamics. At the same time, modeling has progressed such that we are now able to provide more precise predictions on the temporal dynamics for specific systems. This development has reduced the interest in the quite broad predictions provided by the original theories. Nevertheless, most real world systems will never be covered by realistic models and broad generalizations will be valuable for predicting the dynamics in these systems. For instance, the dynamics of terrestrial arthropod populations are seldom studied across gradients of nutrient enrichment at a scale where population level mechanisms become relevant, and at the same time, destabilizations due to nutrient enrichment are often implied in various studies involving pest insects [4].

Islands with nesting seabirds provide one potential way to study effects of increased nutrient availability on arthropod population dynamics at a population level. Seabird nesting colonies often occur on islands and their feces often affect nutrient availability for plants across the whole island. Consequently, the effect on arthropod numbers and densities will occur through population level mechanisms rather than through movement processes, where individuals would congregate in resource rich areas. Moreover, the range of nutrient availabilities for islands with and without nesting seabirds is larger than is typically achieved in traditional fertilization experiments. In extreme cases, nutrient loads are so high that plant growth is compromised due to ammonium toxicity. A drawback from using seabird islands is that the treatment (fertilization) is not tightly controlled. However, by working along a range of seabird nesting activity, we believe that realistic effects from increased nutrient availabilities on plant-insect interactions can be observed. The natural experiment provided by seabird defecation provides an opportunity to test fertilization effects at a scale where traditional experiments are not possible.

**Figure 1:**
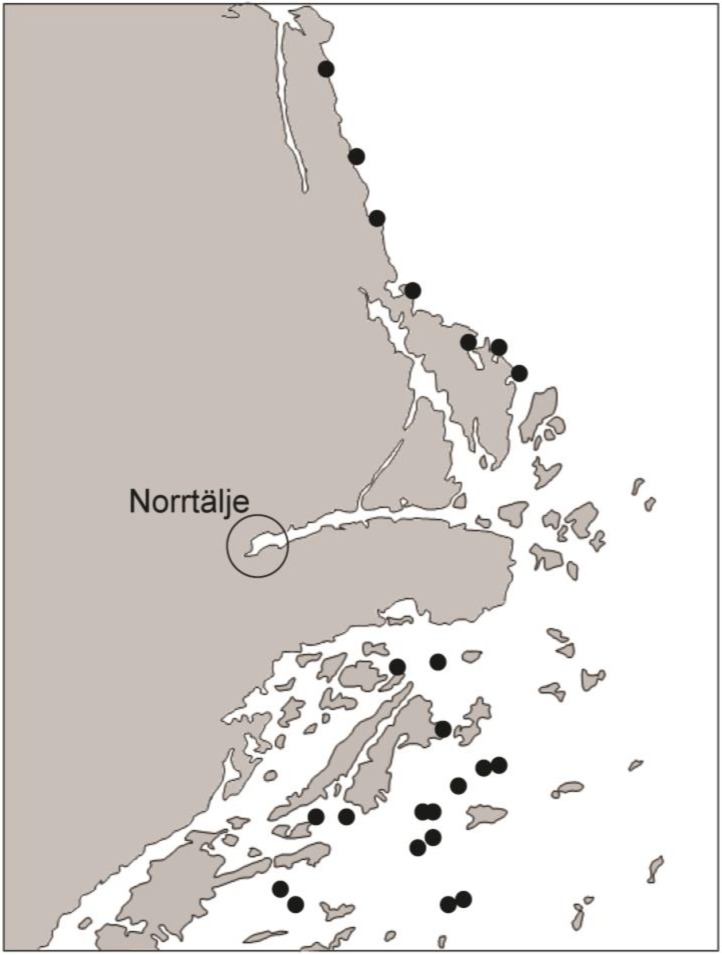
Map of the study area. Solid dots indicate sampling sites.

In this study, we used the plant *Lythrum salicaria* and its associated herbivore fauna (*Galerucella* spp., *Chrysomelidae*) to quantify effects of increased soil nutrients on plant nutrient availability, herbivore density and herbivore dynamics, on shores and islands in the Stockholm archipelago (Figure 1). Unfortunately, we were unable to separate two closely related *Galerucella* species (*G. pusilla* and *G. calmariensis*) and the densities and dynamics are therefore joint measures of two species. Other studies in the same area, however, show that *G. calmariensis* is by far the most common species in coastal sites, typically more than 80%. In a pot experiment, we also experimentally quantified stoichiometric variation in the herbivores and their performance along a gradient of plant nutrient content to relate the dynamic responses to theories of ecological stoichiometry (ES). The general pattern for heterotrophic organisms is that N and P contents show little variation with food nutrient content. ES suggests that such homeostasis may cause individual growth and reproductive rates to decrease when the resource stoichiometry deviates from the consumer stoichiometry. For this system, we know that pupal mass affects female fecundity in the following years suggesting a potential link between plant nutrient content, individual growth rates and population growth rates depending on the relationship between plant nutrient content and pupal mass.

## 2. Study Organism

Purple loosestrife, *Lythrum salicaria* L. (*Lythraceae*), is a perennial, insect-pollinated herb that is native to Eurasia and has been introduced to North America [12]. It is abundant on islands and the shore-line in most areas of the northern Baltic Sea. Reproducing plants are on average 50 cm tall and produce one to several flowering shoots. Flower buds develop in leaf nodes in the upper part of the flowering shoot. In Fennoscandia, *L. salicaria* flowers for six to eight weeks in July–August. The seeds mature six to eight weeks after flowering.

The beetles *Galerucella calmariensis* L. and *G. pusilla* L. (*Coleoptera*: *Chrysomelidae*) are monophagous herbivores on *L. salicaria* [13], feeding on leaves and flower buds. Their life cycle is univoltine and the adults overwinter in the litter [14]. Around Stockholm, adults appear in May when the first host plant leaves appear. The adults feed on leaves and lay their eggs in batches on the stem and on the lower leaf surface. The larvae hatch seven to 10 days later, feed on leaves and flower buds for two to three weeks and then pupate in the ground. Previous studies suggest that larval growth is limited by both temperature and intraspecific density [15,16]. Similar to several other herbivore systems [17,18], low larval densities reduce pupal mass and thereby also reduce fecundity of females in the next generation.

## 3. Results

### 3.1. Field Study

**Figure 2:**
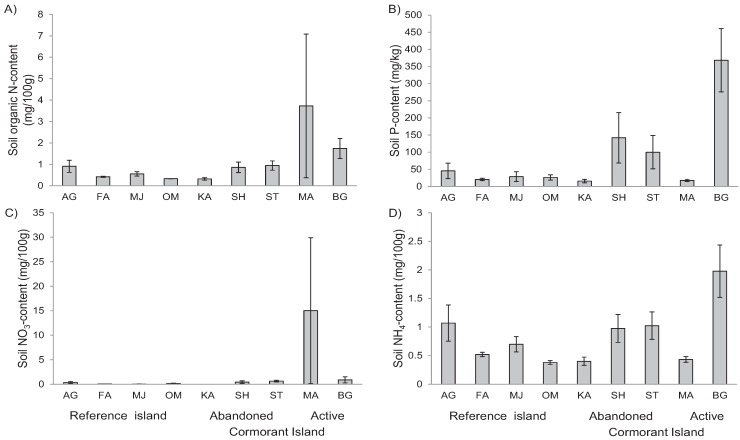
Soil nutrient contents (N, P, NO_3_, and NH_4_) on four non-cormorant, three abandoned and two active cormorant islands in Stockholm archipelago.

**Figure 3:**
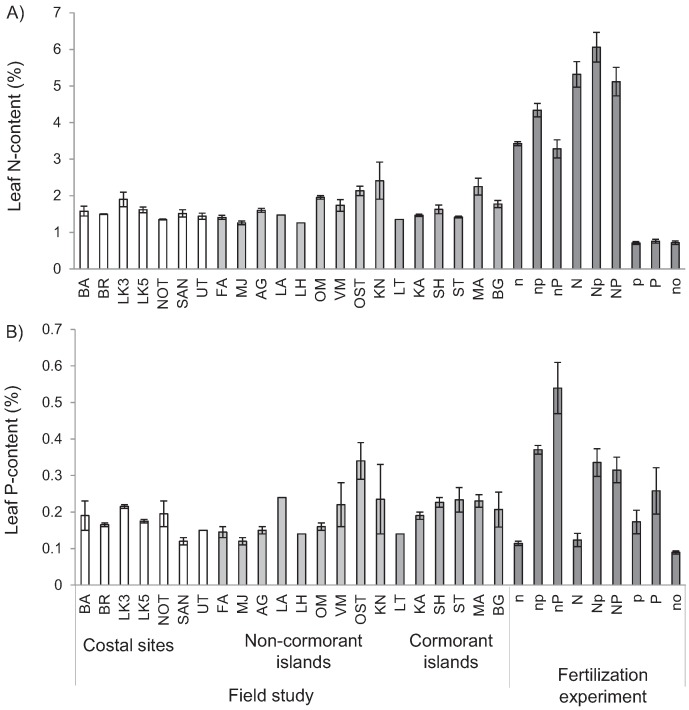
*Lythrum salicaria* leaf N- and P-contents collected on field costal field sites, non-cormorant islands, abandoned and active cormorant islands and from fertilization pot experiment (medium (n and p) hand high (N and P) level of N and P fertilization and no fertilization (no)).

The soil nutrient content showed strong variation among islands. Total soil N-content was higher on active cormorant islands than on non-cormorant islands (F_2.6_ = 6.8, *p* < 0.05, adj. R^2^ = 59.2%), while soil NH_4_ (F_2.6_ = 1.1, *p* > 0.3), NO_3_ (F_2.6_ = 4.1, *p* = 0.076), and P (F_2.6_ = 0.7, *p* > 0.2) contents did not differ between island categories. The different outcomes of the analyses of total N, NH_4_ and NO_3_ contents occurred because one active nesting island (Marskär, MG) had increased soil NO_3_ while the other active nesting island (Bergskär, BG) had increased soil NH_4_ (Figure 2). Soil P content showed strong variation between islands and was increased on three of five cormorant islands (Figure 2). Leaf nutrient contents (N and P) varied between islands (Figure 3) but did not vary between categories (F = 1.5, *p* > 0.2 and F = 0.9, *p* > 0.3) and were not correlated with soil nutrient content (F = 2.3, *p* > 0.1 and F = 0.2, *p* > 0.6). Plant height did not vary between island categories (F = 1.1, *p* > 0.3).

**Figure 4:**
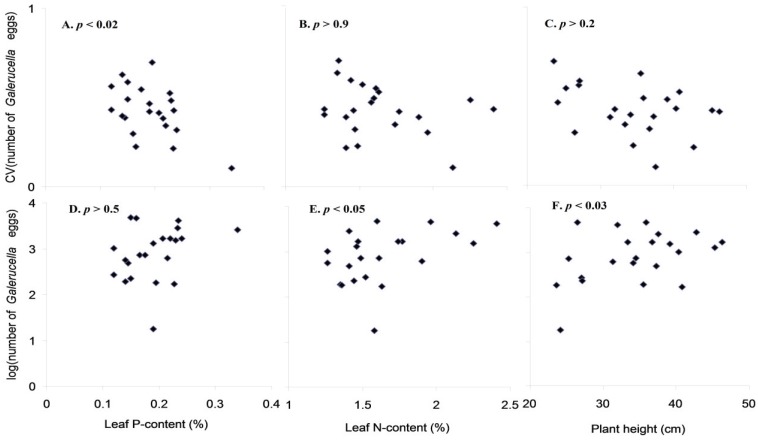
Relationships between *L. salicaria* leaf P-content (**A**,**D**); N-content (**B**,**E**); plant height (**C**,**F**) and mean number of *Galerucella* per plant (**D**–**F**) and the coefficient of variation (CV) of the number of *Galerucella* over four years (a–c).

The mean number of *Galerucella* per plant was positively correlated with leaf N content (F = 5.7, *p* < 0.03) and plant height (F = 5.8, *p* < 0.03) but not with leaf P content (*p* > 0.5) (Figure 4). The CV for *Galerucella* densities across the four years was negatively correlated with leaf P content (F = 7.0, *p* < 0.02) but not with either leaf N content (*p* > 0.9) or plant height (*p* > 0.2) (Figure 4). The correlation with leaf P relied on a low CV for the single sample with very high leaf P content. Excluding this value largely retain the slope (−0.86 *vs*. −1.40) but cause the relationship to be non-significant. We performed similar tests with the number of *Galerucella* per cm plant and these tests provided similar outcomes, probably because plant size did not vary among islands.

### 3.2. Pot Experiment

**Figure 5:**
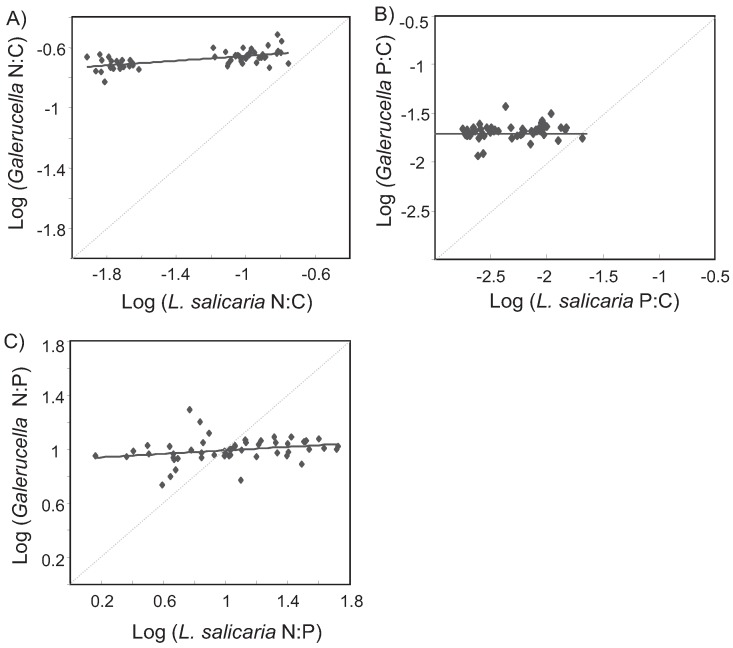
Relationships between elemental ratios of *Lythrum salicaria* and *Galerucella*. The thin line indicates a one-to-one relationship, where plant and beetle elemental ratios are identical.

**Figure 6:**
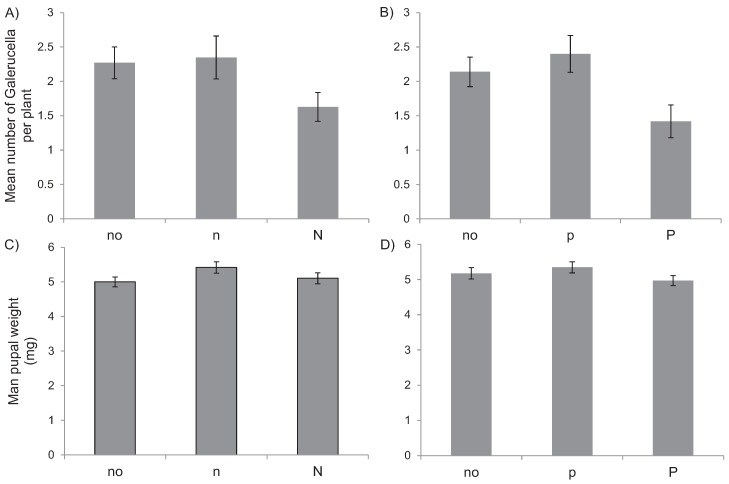
Mean number of *Galerucella* per *L. salicaria* plant (**A**,**B**) and mean pupal weight (**C**,**D**) in different fertilization treatments. No fertilization (no), medium (p) and high (N) nitrogen fertilization (**A**,**C**), and medium (n) and high (P) phosphate fertilization (**B**,**D**).

In the pot experiment, the leaf nutrient content of *L. salicaria* varied strongly with the fertilization treatments (Figure 3; N: *F*_8.57_ = 80.4, *p* < 0.0001, adj. R^2^ = 91%; P: *F*_8.55_ = 16.3, *p* < 0.0001, adj. R^2^ = 66%). Plant N-content varied up to 8.5-fold between treatments, and plant P-content up to six-fold. Plants fertilized with a high nutrient concentration of either N or P generally increased the concentration of this nutrient in their leaves more strongly if they were also fertilized by a low concentration of the other nutrient. *Galerucella* larvae generally regulated their stoichiometry strongly, showing strong N:C (1/H_N:C_ ≈ 0.08) and N:P (1/H_N:P_ ≈ 0.06) homeostasis and strict P:C homeostasis (Figure 5). The survival of *Galerucella* larvae in the experiment was generally low (21%). While N fertilization did not affect the survival, P fertilization showed a weak negative effect on survival (F_2.247_ = 3.2, *p* < 0.05, adj. R^2^ = 1.7%). The mean pupal weight was not affected by fertilization treatments (Figure 6).

## 4. Discussion

Population dynamic responses to nutrient enrichment are rarely observed in the field, because nutrients are seldom manipulated at a scale where population level mechanisms matter. Previous studies therefore mainly involve movement mechanisms where individuals may have aggregated in fertilized plots, rather than being born into them. In this study, we observed density fluctuations of two closely related herbivorous chrysomelid beetles on islands along a natural gradient in soil nutrient levels caused by defecation from nesting cormorants and roosting seabirds. These islands varied in soil nutrient contents, with an increased N content on active nesting islands and an increased P content on three out of five nesting islands (see also Ref. [19]). Surprisingly, the variation in soil nutrient content did not translate into a corresponding variation in leaf N and P contents, contrary to previous measures on the same islands for a broader set of plant species [5]. Nevertheless, the islands showed substantial variation in leaf nutrient contents and the *Galerucella* number was in fact correlated with both leaf N content and plant height. On the other hand, and contrary to the expectation, the variability in *Galerucella* densities did not increase with either leaf N or P contents. In fact, the coefficient of variation in *Galerucella* densities tended to decrease with increased leaf P content. This effect is not likely due to a too short time series, as the pattern of fluctuations on control sites is similar to 13–15 year long time series collected from other coastal sites (Hambäck unpublished data). It should be remembered that densities are joint measures of two *Galerucella* species and therefore that different dynamics in the two species may have affected the result. We cannot exclude this possibility, but note that one species (*G. calmariensis*) normally dominates coastal sites in the area (>80%). 

Nitrogen limitation and density responses to increased N are commonly observed in arthropod populations [3,6], whereas P limitation is more seldom observed, similar to our study. The mechanism underlying the observed response is less clear and the pot experiment provided no further information as pupal weights and survival did not respond to the nitrogen fertilization treatments, suggesting that other growth stages or interactions with other organisms may have been more important in the field. Previous studies on the same cormorant islands suggest strong responses in some taxa to cormorant nesting but also that some groups increase while other groups decrease (for a similar example in another system, see Ref. [6]). Among herbivore groups, *Lepidoptera* and aphids always had the highest density on active nesting islands, islands where the leaves from a set of collected plant species other than *L. salicaria* had extremely high N and P contents [5]. Herbivorous *Coleoptera*, on the other hand, had higher densities on abandoned islands, where phosphorus but not nitrogen content was increased in the soil, than on active nesting islands. Why some plants increase in N or P content but not others is currently unknown, but may be related to either difference in plant physiological responses or to differences in microsite conditions. Among predatory groups, parasitic *Hymenoptera* had the highest density on active nesting islands while web spiders had the highest density on abandoned nesting islands. In contrast, wolf spiders had the lowest density on active nesting islands. No doubt, responses by specific taxa may depend on responses in other groups and the strength of top-down and bottom-up processes may vary with fertilization [2], and this may underlie the observed responses by herbivores on *L. salicaria*.

The observation of reduced fluctuations in high-P conditions was surprising and contrasted with our expectations, and we are currently unable to explain the underlying mechanism. The pot experiment did indicate that larval survival may decrease with an increased P fertilization, while there was no effect on pupal mass. On the other hand, the leaf P gradient in the pot experiment was much larger than observed in the field, and the reduced larval survival was only observed at the highest fertilization levels. It is also notable that the larvae showed a fairly strict homeostasis in relation to N:C, P:C and N:P ratios, similarly to observations in other terrestrial insect systems [19,20]. An alternative mechanism for the reduced fluctuations in high-P conditions could be that some other life history trait is non-linearly related to leaf P content. If that is the case, a high quality resource may buffer population growth in years when conditions are otherwise poor, e.g., in the case of very dry weather.

To conclude, we found limited support for the suggestion that arthropod populations are destabilized by nutrient enrichment within the *Lythrum-Galerucella* system using islands with and without nesting cormorants. Even though egg densities of *Galerucella* increased in relation to both N content and plant height, fluctuations in density did not increase with a higher N and P. Whether this is a general pattern is unclear, but the results do suggest that increased density fluctuations at a population level should not necessarily be the outcome of fertilizing plants.

## 5. Methods

### 5.1. Field Study

The field study was conducted on islands in the northern part of the Stockholm archipelago and at nearby shore sites on the larger island Väddö, north of Norrtälje (Figure 1). The study sites differed in their input of marine nutrients via nesting and roosting seabirds and shore-drifted algal wrack. In total, 16 islands (2 active cormorant colony islands, 3 abandoned cormorant colony islands, 11 islands without seabird nesting colonies but partly visited by roosting seabirds) and 7 shore sites were included in this study. Additional active cormorant nesting islands occurred in the area, but *L. salicaria* was only found on two such islands. During the end of June and beginning of July 2006–2010, we counted eggs of *G. calmariensis* and *G. pusilla* on up to 56 *L. salicaria* individuals per site, randomly selected along the shore. Because eggs could not be identified to species, we pooled the data for the two *Galerucella* species even though island sites such as these are typically dominated by *G. calmariensis* [21]. We divided the study sites into four categories (shore sites, islands without nesting cormorants, islands with abandoned cormorant colonies and islands with active cormorant colonies). For each site, we calculated the mean number of *Galerucella* per plant and per cm plant. In order to estimate variation in density over the years we calculated the coefficient of variation (CV) defined as the ratio of the standard deviation to the mean. All study sites are sufficiently isolated from each other and from other sites with *L. salicaria* that immigration of *Galerucella* beetles is extremely unlikely. Finally, the weather during May and June may in extreme cases cause mortality of larvae, but mainly in sites away from the shore. We did not specifically look for such mortality, but only the first year (2006) was a dry year during the study period.

#### 5.1.1. Soil and Plant Analyses

To relate soil and plant chemistry, we collected leaf samples from 1–5 randomly selected *Lythrum* plants on all study sites in 2010. On 9 islands distributed along the nutrient gradient, we also collected 3–5 soil samples (500 mL) close to the same plants from which leaf samples were collected. Soil samples were taken down to a depth of 5 cm with a trowel after removal of the litter layer. For P analyses in soil, samples with dominantly organic material were milled in a cyclotech mill to 0.5 mm, and sandy samples were homogenized in a machine mortar for maximum 3 minutes. Inorganic P was determined following extraction with 2% citric acid (1:5 soil to extract solution) [22]. For ammonium and nitrate analysis, sieved soils were extracted with 2 M KCl (100 g soil per 250 mL liquid) for 2 h for sandy samples and overnight for clayey soils. Soils were filtered and analyzed with flow injection analysis (Foss, Sweden), following the application notes AN 50/84 [23] and ASN 50-01/92 [24].

Before analysis, the leaves were dried at 55 °C to constant weight, ground and subsamples were used for the analyses (P: 3–4 mg, CN: 1–3 mg). Phosphorus content (%P, dry mass basis) was assayed using persulfate digestion and ascorbate-molybdate colorimetry [25]. Nitrogen and carbon content (%N, %C, dry mass basis) was assayed in parallel to stable isotopes in an Isotope Ratio Mass Spectrometer type *Europa integra* or an elemental analyzer. In this analysis, samples were oxidized and reduced to CO_2_ and N_2_, respectively, which were measured with a thermal conductivity detector and IR-detection.

#### 5.1.2. Statistics

In order to test if the soil nutrient contents differed between island categories, we compared soil nutrient content of P (mg/kg dry soil), N, NO_3_, and NH_4_ (mg/100 g dry soil) with an ANOVA. We similarly compared leaf N- and P-content and plant height between the four study site categories with an ANOVA. We used linear mixed effect models to relate soil and plant nutrient contents, using island as a random effect. Finally, we tested if plant nutrient contents (N and P) and plant height affected the *Galerucella* density and its variation over the years by regressing these plant parameters with the mean and CV of *Galerucella* egg number per plant. We also included island size and the size of the *L. salicaria* populations, but neither factor was found to influence either the *Galerucella* density or CV.

### 5.2. Pot Experiment

To study the effect of P and N on *Galerucella* stoichiometry and performance, we conducted a pot experiment in a common garden from May to August 2009. In this study, we used individual *L. salicaria* brought up from seed and potted in commercial soil-sand mixture (3:1), split into 9 treatments (= 25 plants per treatment, for a total of 225 plants). Fertilization treatments had different levels of N (H_4_N_2_O_3_) or P (Na_2_HPO_4_), to achieve at least as wide a gradient in soil and leaf nutrient content as in the field plots (Appendix Table A1). During a 3-month period, plants were watered once a week with dissolved nutrients, starting 4 weeks before the first beetle larvae were placed on the plant and ending when the beetle pupae were collected. In order to quantify the effect from fertilization on plant nutrient content, two leaves of the host plant were collected at the start of the experiment and analyzed for N, P and C content as above. Newly hatched *Galerucella* larvae were placed on the plant (15 per plant), and pupae were collected 4–5 weeks later. The pupae and beetles were directly transferred into plastic tubes and frozen until further preparation. Insect samples were weighed and then freeze-dried. For each treatment, we analyzed 5–7 *Galerucella* pupae and one *Lythrum* leaf for P, C and N. Leaf-nutrient analysis was conducted in the same way as described for the field experiment. Beetles were freeze-dried, lightly crushed and subsamples (P: 2–3 mg, CN: 1–2 mg) were assayed for P, C and N contents as described for leaves.

#### Statistics

We tested the effect of fertilization on leaf nutrient content by comparing leaf N and P contents between the 9 treatments with ANOVA.

To examine the relationship between *Galerucella* and *L. salicaria* stoichiometry we first calculated the homeostasis coefficient *H*:

(1) $$H=\frac{\log\left(x\right)}{\log\left(y\right)-\log\left(c\right)}$$

where *x* is the resource elemental mass ratio (*N*:*C*, *P*:*C*, *N*:*P*), *y* is the consumer elemental ratio and *c* is a constant [26]. Equation 1 can be linearized as

(2) $$\log\left(y\right)=\log\left(c\right)+\frac{\log\left(x\right)}{H}$$

such that the degree of homeostasis can be found in a linear regression between the logarithms of the consumer and resource elemental ratios. A given taxon (*Galerucella*) is defined to be strictly homeostatic if its stoichiometry is tightly constrained across a wide variation in resource (*L. salicaria)* stoichiometry [26]. The slope of the regression line (1/H) describes the strength of consumer homeostasis. For each plant we calculated the mean pupal weight and tested if nitrogen or phosphorus fertilization affected the survival and weight of *Galerucella* with ANOVA. All analyses were conducted in the free software R 2.12.1.

## 6. Conclusions

This study examined the effect of fertilization from cormorant defecation on the abundance and dynamics of herbivores on purple loosestrife (*L. salicaria*). Even though we found higher soil N and P contents on cormorant nesting islands, plant leaves had no higher nutrient contents on these islands. Plant N content was still a good predictor of herbivore abundance, and the number of *Galerucella* eggs increased with N content. There was also a tendency that the variation of egg number varied with P content, but the variation was lower on islands with a higher leaf P content. We thus found limited support for the suggestion that arthropod populations are destabilized by nutrient enrichment within the *Lythrum-Galerucella* system.

## Appendix

**Table A1:** Fertilization treatments for *Lythrum salicaria* in a pot experiment in the common garden. Per treatment we used 25 plants. Plants were watered once a week with 1 L nutrient solution.

	n	np	nP	N	Np	NP	p	P	no
Na_2_HPO_4_ (g/L)	0	0.94	2.51	0	0.94	2.51	0.94	2.51	0
H_4_N_2_O_3_ (g/L)	3.34	3.34	3.34	8.91	8.91	8.91	0	0	0

## References

[1] Schoonhoven L.M., van Loon J.J.A., Dicke M. (2005). Insect-Plant Biology.

[2] Gratton C., Denno R.F. (2003). Seasonal shift from bottom-up to top-down impact in phytophagous insect populations. Oecologia.

[3] Haddad N.M., Haarstad J., Tilman D. (2000). The effects of long-term nitrogen loading on grassland insect communities. Oecologia.

[4] White T.C.R. (1993). The Inadequate Environment.

[5] Kolb G.S., Jerling L., Hambäck P.A. (2010). The impact of cormorants on plant-arthropod food webs on their nesting islands. Ecosystems.

[6] Meyer G.A., Root R.B. (1996). Influence of feeding guild on insect response to host plant fertilization. Ecol. Entomol..

[7] McCauley E., Nisbet R.M., Murdoch W.W., de Roos A.M., Gurney W.S.C. (1999). Large-amplitude cycles of *Daphnia* and its algal prey in enriched environments. Nature.

[8] Rosenzweig M.L. (1971). Paradox of enrichment: destabilization of exploitation ecosystems in ecological time. Science.

[9] Roy S., Chattopadhyay J. (2007). The stability of ecosystems: A brief overview of the paradox of enrichment. J. Biosci..

[10] Arditi R., Ginzburg L.R. (1989). Coupling in predator-prey dynamics: Ratio-Dependence. J. Theor. Biol..

[11] Gurney W.S.C., Nisbet R.M. (1975). The regulation of inhomogeneous populations. J. Theor. Biol..

[12] Hultén E., Fries M. (1986). Atlas of North European vascular plants north of the tropic of cancer. I-III.

[13] Hambäck P.A., Ågren J., Ericson L. (2000). Associational resistance: Insect damage to purple loosestrife reduced in thickets of sweet gale. Ecology.

[14] Hight S.D., Blossey B., Laing J., Declerck-Floate R. (1995). Establishment of insect biological control agents from Europe against *Lythrum salicaria* in North America. Environ. Entomol..

[15] Hambäck P.A. (2010). Density-dependent processes in leaf beetles feeding on purple loosestrife: Aggregative behaviour affecting individual growth rates. Bull. Entomol. Res..

[16] Stenberg J.A., Hambäck P.A., Ericson L. (2008). Herbivore-induced “rent rise” in the host plant may drive a diet breadth enlargement in the tenant. Ecology.

[17] Clark B.R., Faeth S.H. (1997). The consequences of larval aggregation in the butterfly *Chlosyne lacinia*. Ecol. Entomol..

[18] Wise M.J., Kieffer D.L., Abrahamson W.G. (2006). Costs and benefits of gregarious feeding in the meadow spittlebug, *Philaenus spumarius*. Ecol. Entomol..

[19] Kolb G., Palmborg C., Hambäck P.A. (2013). Ecological stoichiometry and density responses of plant-arthropod communities on cormorant nesting islands. PLOS ONE.

[20] Kagata H., Ohgushi T. (2006). Nitrogen homeostasis in a willow leaf beetle, *Plagiodera versicolora*, is independent of host plant quality. Entomol. Exp. Appl..

[21] Hambäck P. (2004). Why purple loosestrife in sweet gale shrubs are less attacked by herbivorous beetles? (In Swedish with English abstract). Entomol. Tidskr..

[22] Hesse P.R. (1971). A Textbook on Soil Chemical Analysis.

[23] (1984). Application Note (AN 50/84). Determination of Ammonia Nitrogen by Flow Injection Analysis and Gas Diffusion.

[24] (1992). Application Sub Note (ASN 50-01/92). Ammonia.

[25] Clesceri L.S., Greenberg A.E., Eaton A.D. (1998). Standard Methods for the Analysis of Water and Wastewater.

[26] Sterner R.W., Elser J.J. (2002). Ecological Stoichiometry: The Biology of Elements from Molecules to the Biosphere.

